# Synthesis and characterisation of cationic quaternary ammonium-modified polyvinyl alcohol hydrogel beads as a drug delivery embolisation system

**DOI:** 10.1007/s10856-015-5637-6

**Published:** 2016-01-19

**Authors:** Clare L. Heaysman, Gary J. Phillips, Andrew W. Lloyd, Andrew L. Lewis

**Affiliations:** School of Pharmacy and Biomolecular Sciences, University of Brighton, Moulsecoomb, Brighton, BN2 4GJ UK; Biocompatibles UK Ltd, Farnham Business Park, Weydon Lane, Farnham, Surrey, GU9 8QL UK

## Abstract

To extend the platform of clinically utilised chemoembolic agents based on ion-exchange systems to support the delivery of anionic drugs, a series of PVA-based beads was produced with different levels of (3-acrylamidopropyl)trimethylammonium chloride (APTA) in their formulation. The beads were characterised to confirm composition and the effect of formulation variation on physical properties was assessed. Suspension polymerisation was shown to successfully produce uniformly spherical copolymer beads with APTA content up to 60 wt%. Equilibrium water content and resistance to compression both increased with increasing APTA content in the formulation. Confocal laser scanning microscopy was used with model drugs to demonstrate that by increasing APTA content, compounds between the molecular weight range 70–250 kDa could permeate the microsphere structures. Interaction with anionic drugs was modelled using multivalent dyes. Dyes with multi-binding sites had increased interaction with the polymer, slowing the release and also demonstrating a reduced rate of elution from beads with higher charge density. The model drug release studies demonstrate that these systems can be engineered for different potential anionic drugs for local therapeutic delivery in the clinic.

## Introduction

Ion-exchange is the interchange of ions in a liquid phase with counter ions of a solid ionic polymer (resin) and is commonly utilised in drug delivery systems as a method of loading and subsequently controlling the release of a charged drug [1]. Exchange is controlled by factors such as pH, temperature, ionic strength and drug properties such as molecular weight and charge density. Rates of release can be further controlled by altering the polymer structure, modifying the degree of cross-linking or by applying a membrane to encase the resin [2].

We have previously reported on the development of a commercial drug delivery embolisation system known as DC Bead™ which is a cationic exchange system formed by the copolymerisation of a modified polyvinyl alcohol (PVA) macromer and an acrylamido sulfonate monomer [3–6]. In this system ion-exchange occurs between the sulfonate moieties of the copolymer and the protonated primary amine of drugs such as doxorubicin hydrochloride salt. The beads are easily loaded by immersion in an aqueous drug solution and have demonstrated greater than 90 % loading efficiency of doxorubicin [6] in which the drug is stable for at least 14 days when refrigerated [7].

**Fig. 1:**
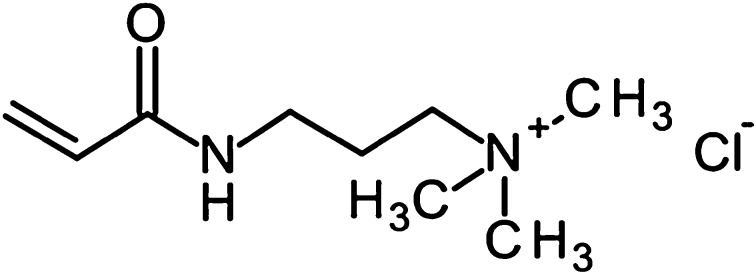
Chemical structure of APTA

There have previously been a number of studies which have considered the synthesis of cationic polymers for the delivery of anionic drugs using the cationic quaternary monomer (3-acrylamidopropyl)trimethylammonium chloride (APTA, Fig. 1) [8, 9]. The aim of this study is to expand on the current platform of chemoembolization bead technologies used in the clinic to enable the delivery of other drugs. Herein we describe the preparation and characterisation of a series of microspheres by directly substituting the negatively charged sulfonate based sodium salt (AMPS) used in the preparation of DC Bead™ with APTA.

## Materials and methods

### Materials

Polyvinyl alcohol (PVA) partly saponified, 88 % hydrolysed, 12 % acetate content, average molecular weight of 67,000 Da (trade name Mowiol 8-88) was supplied by IMCD UK Ltd, Surrey, UK. (3-acrylamidopropyl)trimethylammonium chloride (APTA) (75 % w/w) was supplied by Sigma-Aldrich, UK. *N*-acryloyl-aminoacetaldehyde dimethyl acetal (NAAADA) and 10 % cellulose acetate butyrate (CAB, Safic Alcan UK Ltd, Warrington) solution in ethyl acetate. Pyrene sulfonic acid based sodium salts P1, P2, P3 and P4 were supplied by Sigma Aldrich, UK. Water was purified by reverse osmosis using a Millipore (UK) Ltd Elxi 10 water purification system. Sodium chloride was supplied by Fisher Scientific UK. Phosphate buffered serology saline (PBS) was supplied by Inverclyde Biologicals, UK. Solvents were supplied by Romil Ltd. UK and all other materials were supplied by Sigma-Aldrich, UK and were used as received.

### Macromer and bead synthesis

PVA (300 g) was dissolved in 2L of purified water with heating to 95 °C for 2–3 h. NAAADA (4.98 g) was then added to the PVA solution followed by the addition of concentrated hydrochloric acid (238 g), which catalysed the addition of the NAAADA to the PVA backbone through the acetal groups at 40 °C. After 1 h the solution was neutralised by the addition of 2.5 M NaOH solution to achieve pH 7.4. In this acid catalysed reaction, the cyclic acetal of the macromer may be formed through the removal of methanol and reaction with the pendant 1,3-diols of PVA; it is also likely that in the presence of water, the acid will also act to hydrolyse the NAAADA. This leads to the formation of the aldehyde and subsequently its conversion, through the formation of hemiacetal, to the cyclic acetal of the macromer.

Insoluble beads were produced by water in oil suspension polymerisation where the macromer was copolymerised with APTA by redox-initiated polymerisation using the following phase systems: *Organic Phase:* 600 g *n*-butyl acetate and 11.5 g of a 10 % (w/w) CAB in ethyl acetate were added to a glass 1 L jacketed vessel connected to a heater-chiller unit and stirred at approximately 300 rpm at 25 °C and purged with N_2_ (g). *Aqueous phase:* A known amount of PVA macromer (21 g non-volatile weight), 1.3 g APS, the appropriate amount of APTA solution and an additional amount of purified water were mixed together and added to the reaction vessel. Water was added so that the total amount of water in the formulation was approximately 130 g. Polymerisation was activated through the addition of 1.6 mL tetramethylethylenediamine (TMEDA). An excess amount of TMEDA to APS was used to ensure complete reaction of APS. The reaction was allowed to continue for 3 h at 55 °C under an inert N_2_ (g) atmosphere. The beads were then purified by washing in ethyl acetate and acetone to remove residual CAB, before hydration and washing in water. The beads were heat extracted by boiling in 80 mM disodium hydrogen phosphate in 0.29 % (w/w) NaCl solution before rehydration in water, followed by equilibration in saline. Beads were produced in various sizes, typically 100–1500 µm.

Formulation of the aqueous phase was varied to produce beads with different concentrations of APTA. In all formulations the total amount of water, macromer and APS remained the same. Seven batches of beads were produced using different formulations as outlined in Table 1. Notation for the formulations represents the ratio of weight percentage (wt%) for APTA to macromer used in synthesis e.g. APTA_43_ denotes 43 wt% APTA: 57 wt% macromer. APTA_0_ are beads composed of solely the cross-linked PVA macromer; APTA_100_ was prepared using APTA alone and an additional non-ionic acrylamide monomer (*N*,*N*′-methylenebisacrylamide) in order to provide cross-linking and prevent dissolution of the beads.

**Table 1:** Weight percentage (wt%) of APTA versus macromer in bead formulations

Formulation	APTA (wt%)	Macromer (wt%)
APTA_0_	0	100
APTA_16_	16	84
APTA_27_	27	73
APTA_43_	43	57
APTA_60_	60	40
APTA_86_	86	14
APTA_100_	100	0

### Characterisation of beads

The beads were characterised using image analysis, compression testing and measurement of equilibrium water content as described previously [5, 10, 11].

### Elemental analysis

The weight percentage of carbon, hydrogen and nitrogen in each formulation was determined by elemental analysis. Samples were prepared by dehydrating the hydrated beads of each formulation in acetone and then drying under vacuum at 120 °C. The beads were ground into a powder using a pestle and mortar before final drying to a constant weight. The samples were sent to Medac Ltd, UK for testing. Elemental analysis was performed by combustion testing in the presence of oxygen.

### Image analysis

Optical microscopy imaging was performed using an Olympus BX50F4 microscope equipped with phase contrast filters. Images were captured with a ColorView III camera (Olympus, Japan) which is a high resolution digital camera and was attached to the microscope. Bead sizing was performed manually using the sizing tool of AnalySIS software (Soft Imaging System GmbH).

### Compressibility

Beads used in compression testing were collected from the 710 to 850 µm sieved fraction. Compression testing was performed using an Instron 4411 tensile testing system with Series IX software (Instron, MA) to generate curves of load versus percentage strain. The Young’s modulus of the beads was then determined from the slope of the curve and is an indication of resistance to compression. After the beads were distributed on the testing surface to form a monolayer, residual water was removed by wicking using tissue paper. The beads were compressed using a 5.0 mm^2^ probe attached to a 50 N load cell with a crosshead speed of 5.0 mm min^−1^.

### Equilibrium water content

Equilibrium water content (EWC) of the beads hydrated in water was tested gravimetrically. Excess water was removed from the beads in solution to form a slurry. The beads slurries were then dehydrated in an oven at 120 °C under vacuum until a constant weight was reached. EWC is defined as the weight of dehydrated beads as a percentage of the weight of the initial hydrated slurry.

### Molecular weight cut-off

The molecular weight cut-off was determined as described by Vandenbossche et al. [12]. In summary, the beads from each formulation were imaged in solutions of 10 mg mL^−1^ FITC-Ds of different molecular weights of between 4 and 250 kDa (Sigma-Aldich, UK) using a Leica TCS SP5 confocal, with a Leica DMI6000 B inverted microscope.

### Attenuated total reflectance Fourier transform infrared spectroscopy (ATR-FTIR)

A selection of beads of each formulation were dehydrated and ground to a fine powder using the same method as the samples tested by elemental analysis. The powder was analysed using a diamond crystal on a PerkinElmer Spectrum One FTIR Spectrometer with a Universal Attenuated Total Reflectance (UATR) accessory.

### Loading and elution of model dyes into cationic beads

**Fig. 2:**
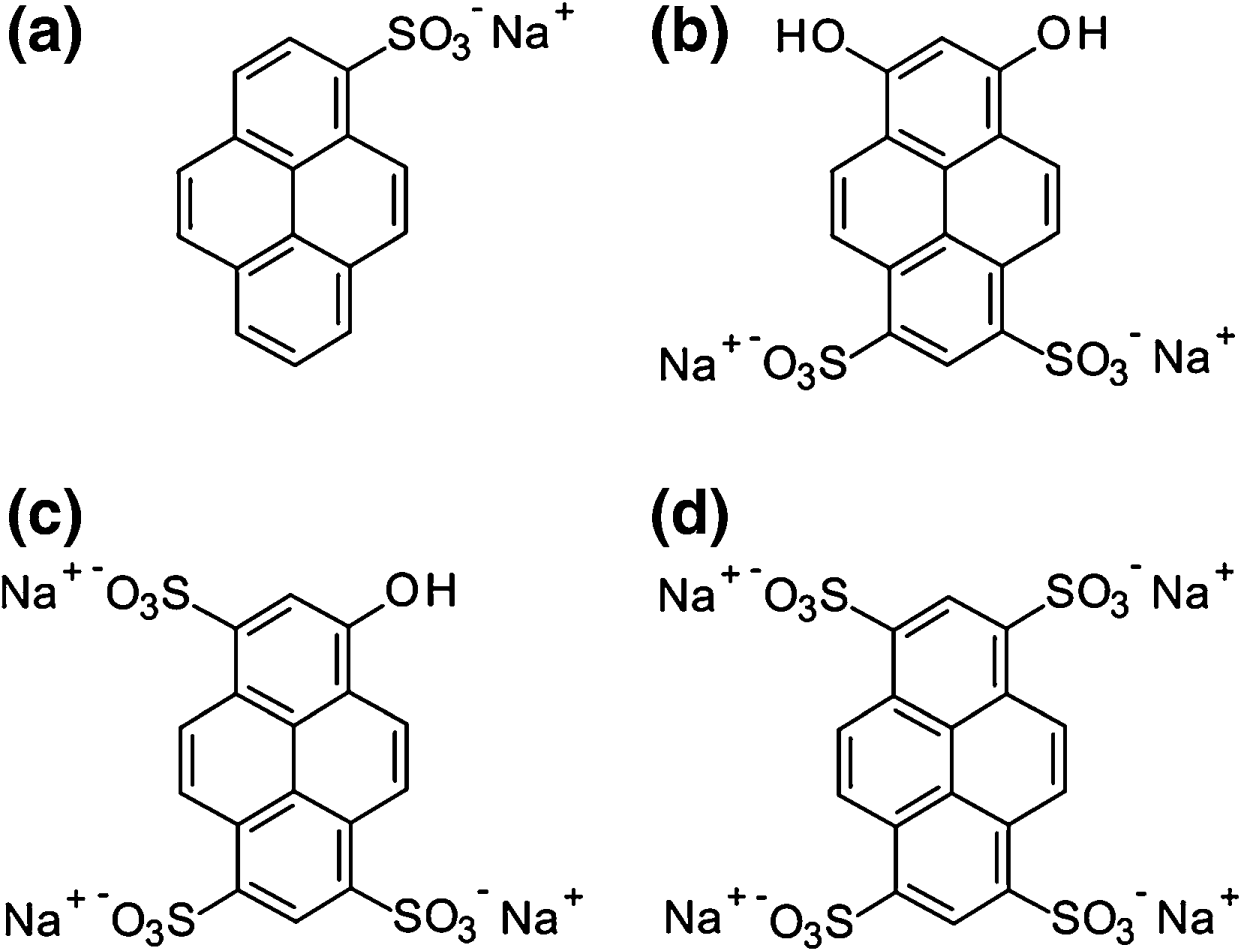
Chemical structures of pyrene sulfonic acid based sodium salts **a** P1, CAS [59323-54-5] **b** P2, CAS [61255-63-8] **c** P3, CAS [6358-69-6] **d** P4, CAS [59572-10-0]

The chemical structures of the model dyes used in this study are shown in Fig. 2; 1-pyrenesulfonic acid sodium salt (P1), 6,8-dihydroxypyrene-1,3-disulfonic acid disodium salt (P2), 8-hydroxypyrene-1,3,6-trisulfonic acid trisodium salt (P3) and 1,3,6,8-pyrenetetrasulfonic acid hydrate tetrasodium salt (P4). The cationic bead formulations selected for this study were collected from the 500 to 710 µm sieved fraction and loaded with dye following the method described by Lewis et al. [6]. Specifically, for each bead formulation a desired volume (e.g. 1 mL) of beads hydrated in saline were measured out using a measuring cylinder. The beads were then transferred to a vial and the saline solution was removed. A dye solution of desired concentration was prepared by dissolving the dye in deionised water. Dye solution was then added to the vial containing the slurry of beads. For the duration of loading the vial was rolled to mix at room temperature. Loading was monitored by removing aliquots of the loading solution. The solutions were analysed by visible spectroscopy using a UV/Vis spectrophotometer Lambda 25 PerkinElmer and UVWinlab software to determine the wavelengths of maximum absorbance at 375 nm for P1, 411 nm for P2, 404 nm for P3 and 376 nm for P4. The amount of dye in solution was determined by visible spectroscopy with reference to standard curves for each dye, prepared in the appropriate experimental media and the amount of dye loaded was calculated from the depletion of the initial solution.

The maximum binding capacity of each formulation with each dye was determined by extraction in an ionic solution. The bead slurries were mixed for 72 h with approximately 5 mg of excess dye per mL of beads, based on calculated theoretical capacities. The beads were then repeatedly washed with purified water to remove residual unbound dye. To extract the dyes the loaded beads were eluted in 500 mL of a saturated KCl solution in water, mixed to a 50:50 ratio with ethanol. The solutions were stirred to mix for 7 h before sampling.

Elution of Dye loaded beads was performed in a fixed volume of PBS and the experiment was performed in triplicate for the formulations APTA_16_, APTA_43_, and APTA_60_. A fixed volume of beads (1 mL in saline) had been previously measured out using a measuring cylinder and loaded with a known amount of dye. The remaining loading solution was removed and the slurry of beads was added to 200 mL of PBS. The experiments were carried out in amber jars. The solutions were rolled to provide continuous mixing for the duration of each experiment. At each time point the eluent was sampled and tested by visible spectroscopy as previously described. The volume of sampled eluent was replaced with fresh PBS to maintain the elution volume.

## Results and discussion

### Bead composition

The results of elemental analysis for the beads prepared in this study are summarised in Table 2. The percentage of hydrogen and nitrogen can be used as a guide to confirm the percentage of conversion of APTA into the final product. There is good agreement between theoretical calculations of hydrogen and nitrogen content (percentages being within 0.5 %) assuming 100 % hydrolysis of the PVA macromer and taking into account some residual solvent contribution in some of the formulations. This indicates that the reaction is facile in all cases and that the target formulations have been achieved.

**Table 2:** Results of elemental analysis for CHN composition of each formulation

Formulation	C	H	N
APTA_0_			
Theoretical %	54.4	9.0	0.3
Measured average (n = 2) %	54.8	9.2	0.2
APTA_16_			
Theoretical %	53.3	9.0	2.8
Measured average (n = 2) %	53.6	9.3	2.4
APTA_27_			
Theoretical %^a^	53.2	9.0	4.2
Measured average (n = 2) %	55.7	9.7	2.6
APTA_43_			
Theoretical %	53.0	9.1	6.1
Measured average (n = 2) %	52.9	9.4	6.0
APTA_60_			
Theoretical %	52.7	9.1	8.3
Measured average (n = 2) %	51.3	9.3	7.9
APTA_86_			
Theoretical %	52.4	9.1	11.6
Measured average (n = 2) %	51.9	9.5	11.1
APTA_100_			
Theoretical %^b^	51.6	9.1	13.6
Measured average (n = 2) %	49.4	9.3	12.7

The theoretical percentages were calculated assuming 100 % conversion of macromer and APTA during synthesis, with incorporation of 5.7 × 10^−3^ mol of (CH_3_)_2_NCH_2_CH_2_N(CH_3_)CH_2_· and ·OSO_3_H free radicals, with the exception of APTA_0_ which assumed incorporation of 1.1 × 10^−3^ mol of (CH_3_)_2_NCH_2_CH_2_N(CH_3_)CH_2_· and ·OSO_3_H free radicals, and that there is 100 % hydrolysis of the PVA macromer during this process. Adjustments were made for residual acetone^a^ and water^b^ that were present and quantified in some samples

**Fig. 3:**
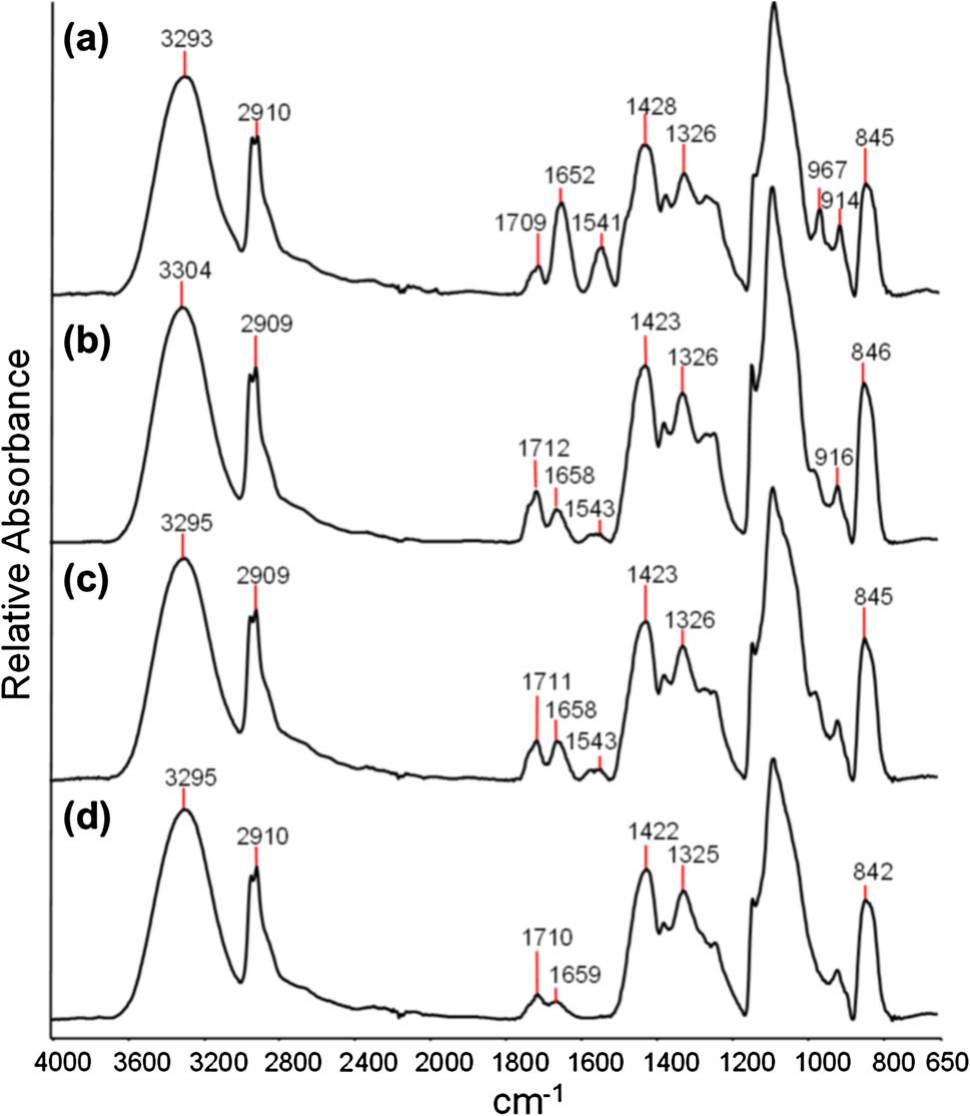
ATR-FTIR spectra of *a* APTA_43_ *b* APTA_27_ *c* APTA_16_ and *d* APTA_0_

The ATR-FTIR spectra of APTA_0_, APTA_16_, APTA_27_, and APTA_43_, are presented in Fig. 3. The spectrum of APTA_16_ is similar to PVA only beads (APTA_0_) with an additional peak at approximately 1545 cm^−1^. This corresponds to the amide II band caused by bending of the amide N–H groups. With increasing concentration of APTA in the formulation the relative intensity of the amide I and amide II peaks appear to increase. In the spectral analysis of formulations APTA_0_, APTA_16_, APTA_27_ and APTA_43_ there is a peak present at approximately 1712 ± 4 cm^−1^. This peak is characteristic of a carbonyl stretching for a ketone group and is due to residual acetone within the beads which was confirmed by GC analysis. The FTIR studies therefore support the elemental analysis in confirming the presence of the appropriate functional groups according to the target formulations.

**Fig. 4:**
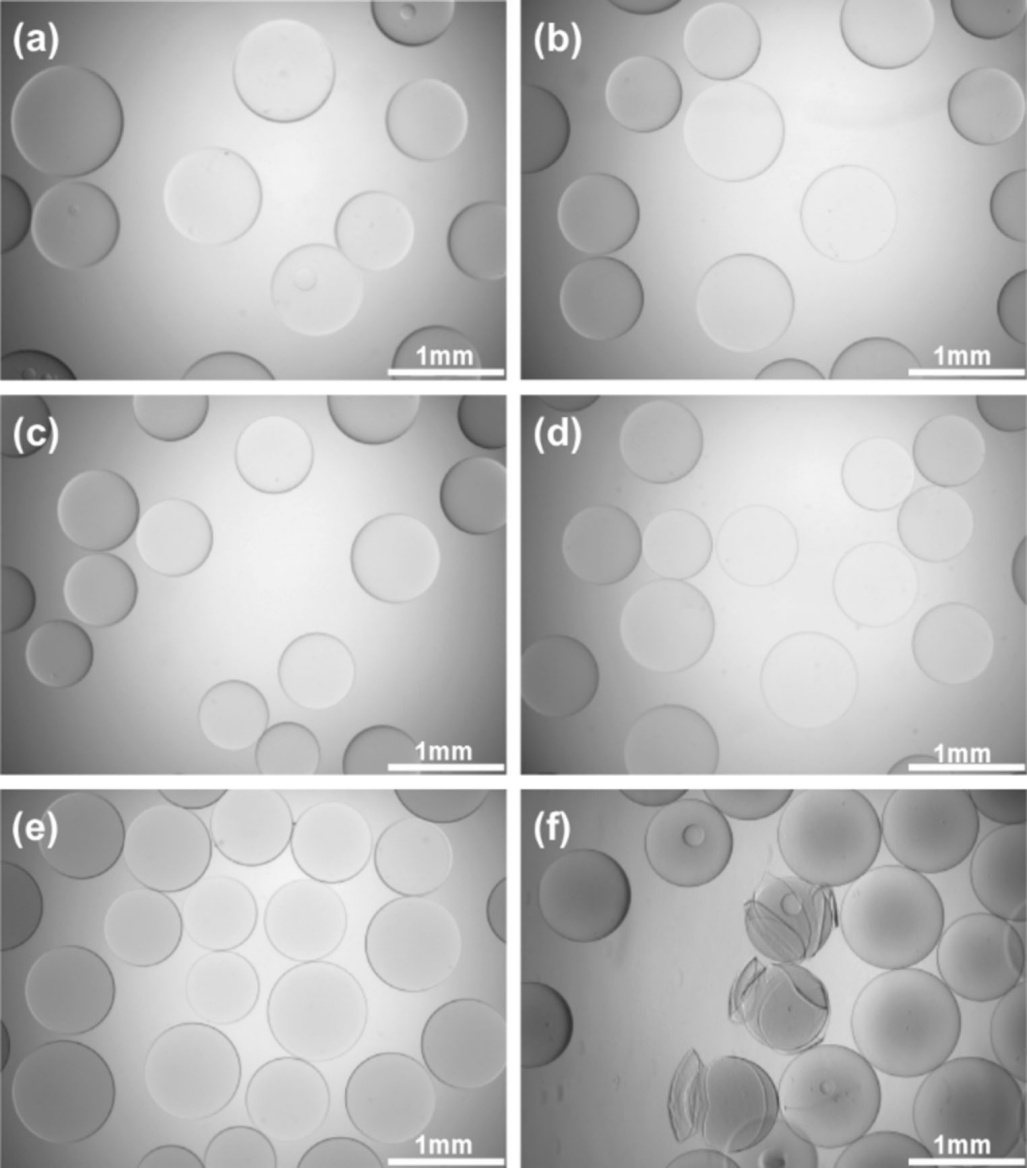
Optical images of beads in saline **a** APTA_0_ **b** APTA_16_ **c** APTA_27_ **d** APTA_43_ **e** APTA_60_ and **f** APTA_86_

Optical images of prepared beads, APTA_0_ to APTA_60_, show that they are spherical and although these beads were not imaged using phase contrast light microscopy, APTA_60_ beads are visibly more opaque than the other formulations (Fig. 4e). Opacity of APTA_60_ beads may be indicative of phase separation. This has previously been observed with the beads containing the anionic acrylamide AMPS when greater than 60 wt% AMPS content is incorporated [13]. As APTA is an analogous cationic acrylamide, a similar affect may be expected for these formulations. From image analysis of APTA_86_ beads it was observed that several appear spherical however there are also many with surface deformities. In Fig. 4f a representative image of APTA_86_ beads is presented. It was observed that some beads appear fragmented with an outer skin peeling away. This was consistently observed in repetitive batches of this formulation.

### Bead properties

**Fig. 5:**
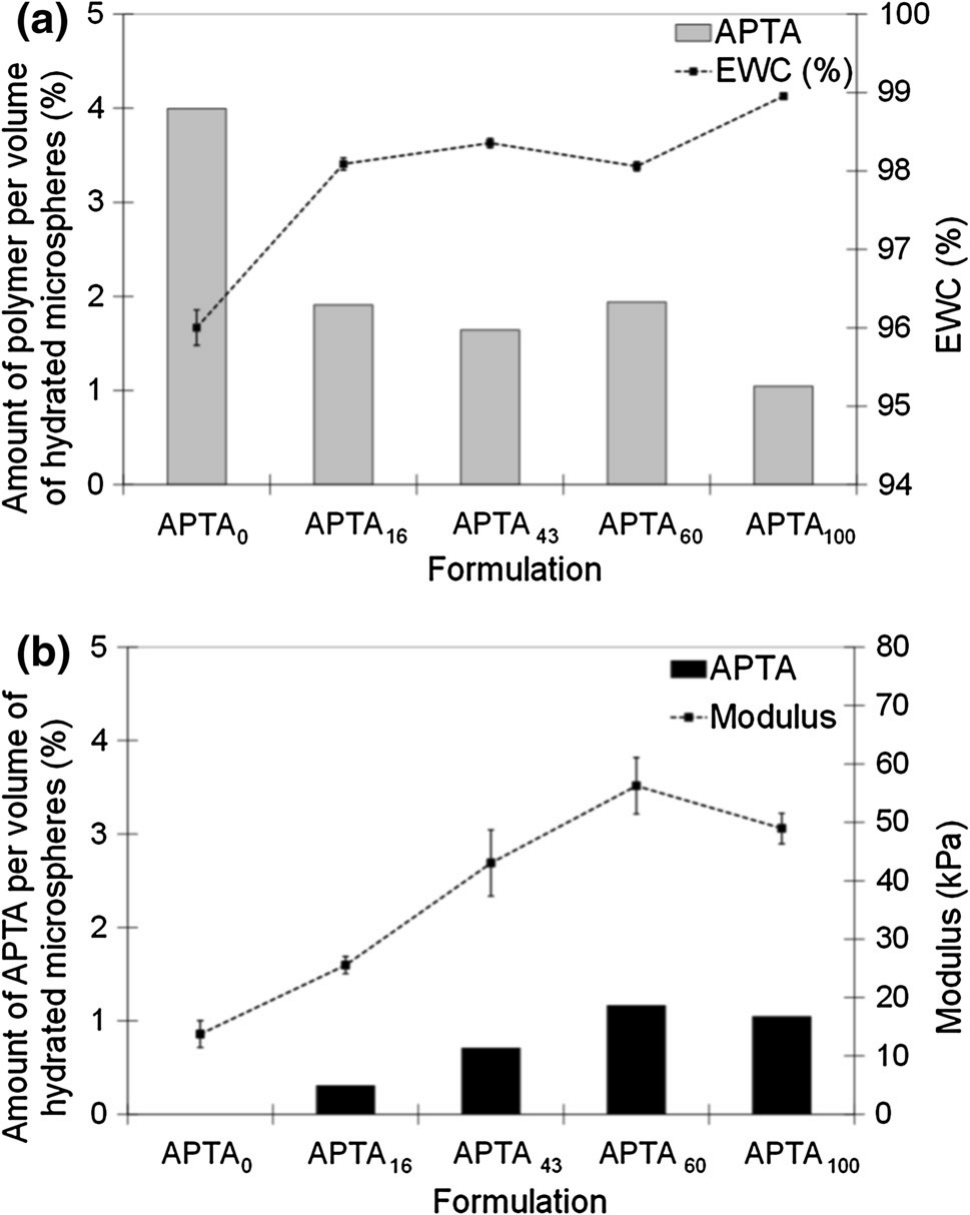
**a** Relative EWC of different APTA bead formulations (mean ± SD, n = 7) **b** Resistance to compression (modulus) of different bead formulations in water (average diameter range 900–1100 µm) (mean ± SD, n = 5)

With increasing APTA content the EWC of the copolymer beads increased (Fig. 5a). With an increase in water content it is assumed that there is a related reduction in the amount of polymer per volume of hydrated beads (Fig. 5b). The ability of the cationic beads to swell is influenced by the elastic nature of the polymer chains. Equilibrium is achieved when the thermodynamic force of swelling equals the constraining force of the polymer chains [14–16]. Although there is a reduction in the total amount of polymer per volume there is an increase in the amount of acrylamide monomer within the polymer fraction. APTA_100_ beads have the highest measured EWC indicating the relative hydrophilicity of the monomer in comparison to the PVA macromer, which is solely present in APTA_0_ beads. Consequently with increasing APTA content the beads become highly swollen and demonstrate increasing resistance to compression.

As these systems possess such high water contents, it is expected that species will be able to freely diffuse into the structure and participate with the functional groups in ion-exchange. The diffusion of FITC-Ds of different molecular weights, into the interior of the hydrogel beads was monitored using confocal laser scanning microscopy to gain some insight into the molecular weight cut off of the beads (essentially the free space between the polymer chain network through which molecules can diffuse). This technique is useful to predict the loading capability of large macromolecules for the prepared bead formulations, as fluorescence can be observed within the centralised regions of the beads if the FITC-Ds are able to diffuse into the structure. A summary of the molecular weight cut-off range, above which FITC-D were not observed in the centre of beads, are summarised in Table 3 and showed that the molecular weight range increased with increasing APTA content, consistent with increasing water content and larger sized water-filled channels within the polymer network.

**Table 3:** Summary of maximum molecular weight cut-off ranges for different cationic bead formulations

Formulation	Molecular weight cut-off range (kDa)
APTA_0_	20–40
APTA_16_	40–70
APTA_43_	70–250
APTA_60_	70–250

### Model pyrene dye loading studies

The theoretical loading capacity for each formulation per volume of beads was calculated by multiplying the theoretical capacity per mg of polymer by the mass of polymer per mL of beads. The maximum binding capacity was measured by complete elution in a saturated ionic solution (Table 4). The theoretical values predict that with increasing APTA in the formulation there is increased loading capability for each dye. This was confirmed as APTA_60_ beads have the highest measured loading capacity with all dyes. The maximum binding capacity of the cationic beads decreased when using dyes of increasing charge density and hence multiple binding points, as these species have the ability to occupy more than one cationic site on the polymer per molecule of dye.

**Table 4:** Theoretical and measured loading capacities of different bead formulations using different dyes P1, P2, P3 and P4 (range, n = 3)

Formulation	Dye	Theoretical bound loading capacity of dye (mg mL^−1^) of beads	Measured bound loading capacity (mg mL^−1^) of beads
APTA_16_	P1	12.1	9.1–9.6
	P2	8.7	10.8–11.2
	P3	7.0	6.3–7.3
	P4	6.1	6.3–6.8
APTA_43_	P1	30.4	22.9–24.1
	P2	21.9	22.8–24.5
	P3	17.5	16.7–18.3
	P4	15.2	17.1–18.4
APTA_60_	P1	41.6	31.2–32.2
	P2	30.0	30.9–33.7
	P3	23.9	21.2–21.5
	P4	20.9	22.1–22.9

**Fig. 6:**
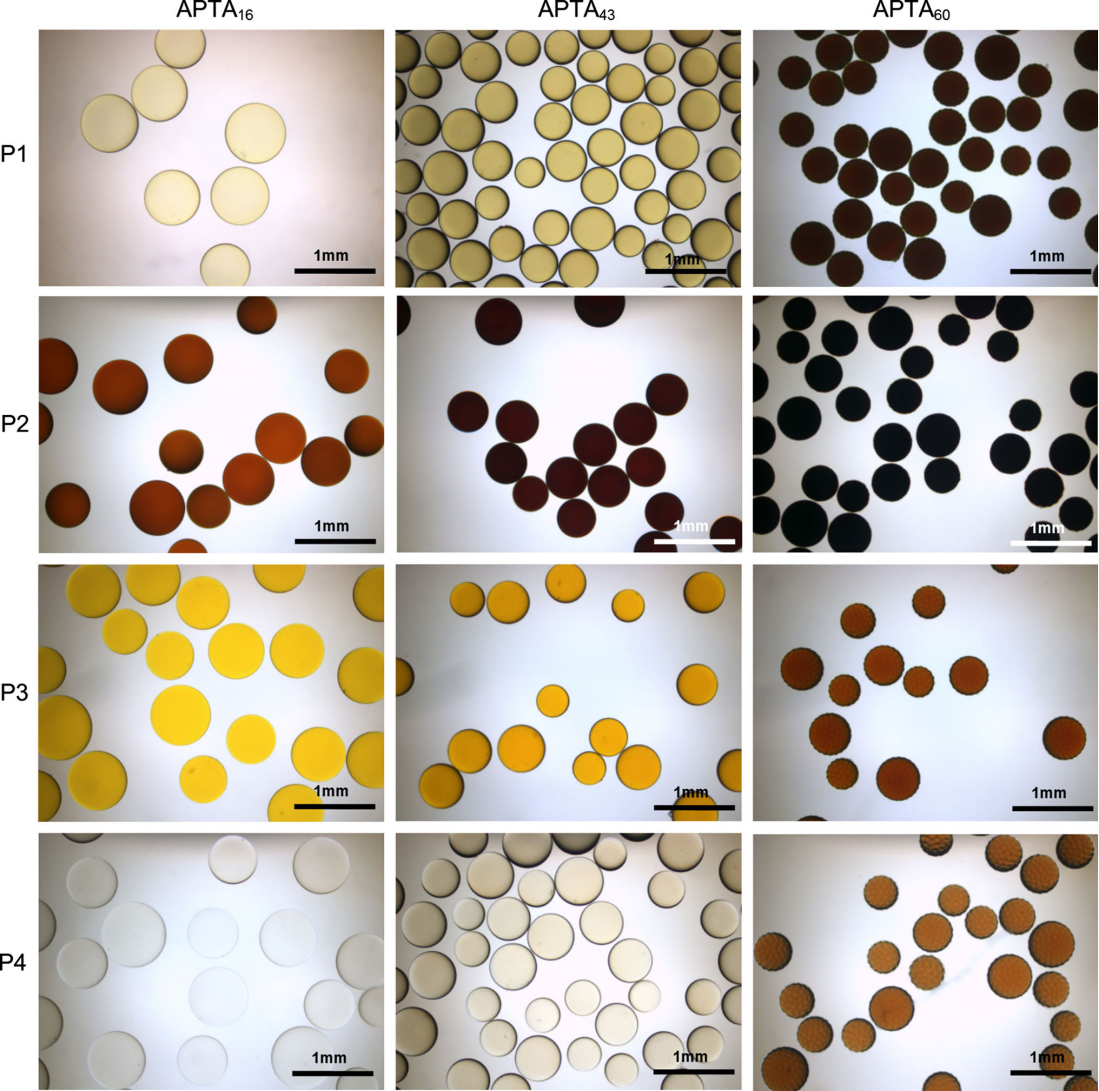
Transmitted light images of cationic beads loaded to maximum capacity with anionic dyes P1, P2, P3 and P4

Transmitted light images of the loaded beads in water demonstrate that they all sequester dye from solution and retain it in their matrix. This is suggestive of an ion-exchange process of loading as there appears to be no diffusion of dye into the surrounding solution. The colour of the loaded cationic beads was dependent upon the type of dye and the amount loaded (Fig. 6).

In Table 5 the average amount of dye loaded per binding site, as calculated from measured values, is presented. It can be concluded that the P1 maximum binding capacity of the beads is less than the theoretical, where for P2, P3 and P4 the amount loaded was as expected given the number of binding sites per molecule.

**Table 5:** Comparative loading capability of different formulations using dyes with different charge densities

Formulation	Average amount of dye loaded per binding site (mol)			
	P1	P2	P3	P4
APTA_16_	0.78	0.63	0.33	0.27
APTA_43_	0.77	0.54	0.33	0.29
APTA_60_	0.76	0.54	0.30	0.27

### Effect of pyrene dye loading on mechanical properties

**Fig. 7:**
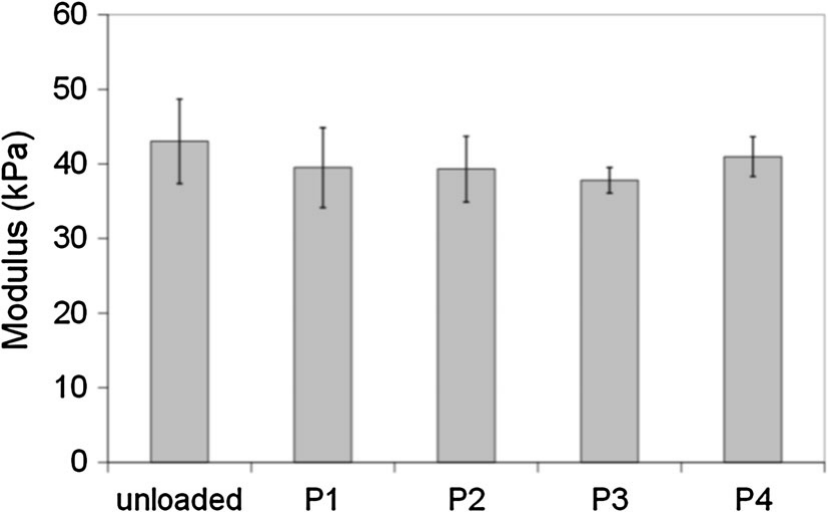
Compression analysis of APTA_43_ beads loaded to maximum capacity with pyrene dyes (mean ± SEM, n = 5)

In Fig. 7 the resistance to compression of APTA_43_ beads loaded to maximum capacity with P1, P2, P3 and P4 is presented. There is no significant difference in the resistance to compression of the beads when loaded to maximum capacity using dyes of varying charge density in comparison to the unloaded beads in water (one-way ANOVA, *P* > 0.05). The measured modulus is not greater than 50 kPa, unlike that observed for doxorubicin interaction with AMPS-based beads [5]; it was suggested that the significant increase in resistance to compression of the anionic beads was due to the behaviour of drug within the bead structure e.g. self-association. In this study there is no evidence to suggest that there is self-association of the dyes as no difference was observed in the resistance to compression of the loaded beads. The modulus of these systems is comparable to other commercially available spherical embolization beads [17] and so may be suitable for delivery through microcatheters and for vessel occlusion.

### Pyrene dye elution studies

**Fig. 8:**
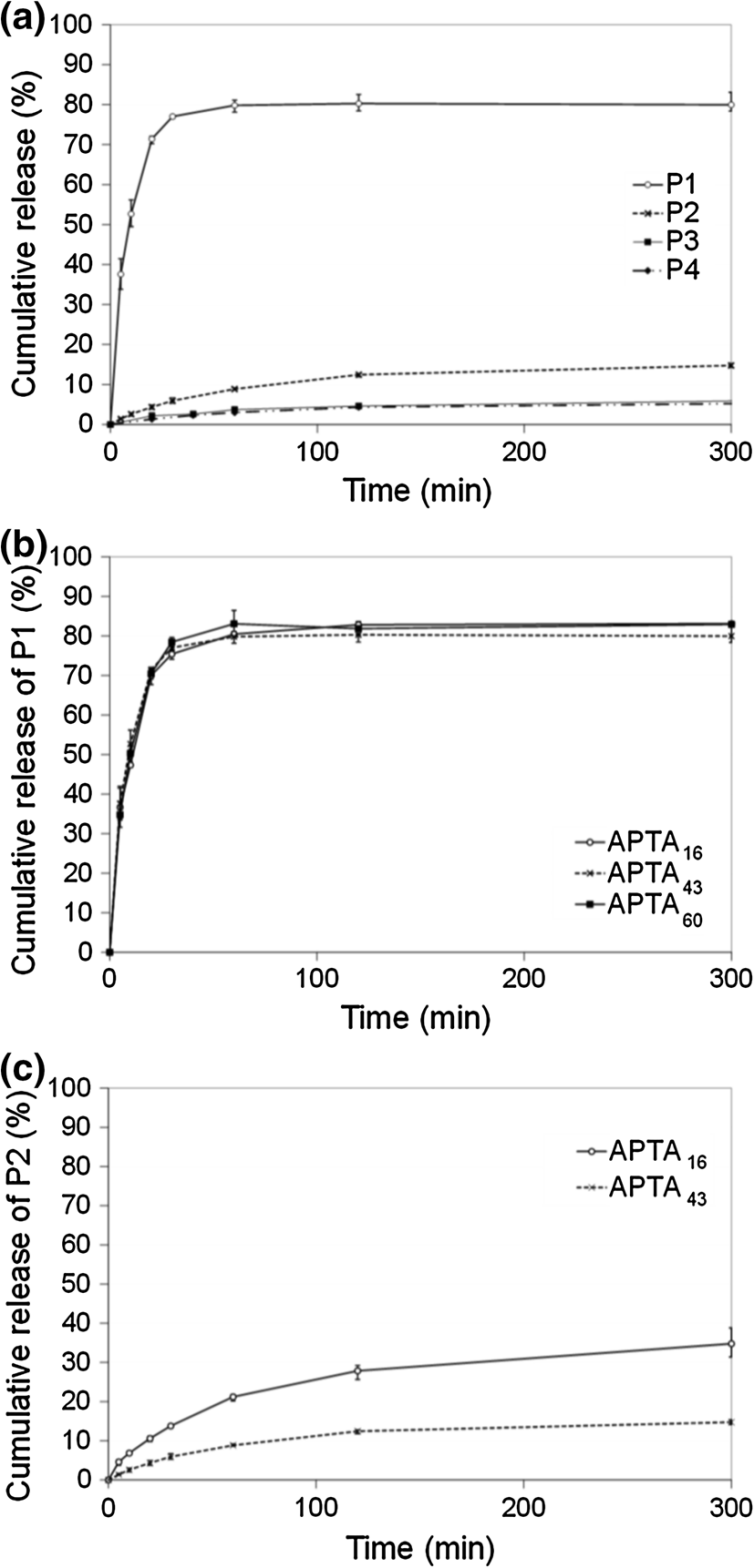
Elution from 1 mL beads in 200 mL of PBS (mean ± range, n = 3) **a** Fraction of P1, P2, P3 and P4 eluted from APTA_43_ beads **b** Fraction of P1 eluted from APTA_16_, APTA_43_ and APTA_60_ beads **c** Fraction of P2 eluted from APTA_16_ and APTA_43_ beads. Initial loading was 8.6 µmol per mL of beads

The elution profiles of P1, P2, P3 and P4 from APTA_43_ beads was assessed and it can be observed that there is a difference in elution rate of the different dyes (Fig. 8a). The monovalent dye P1 has the fastest rate of elution as 80 % of the initial loaded amount was released within 60 min in comparison to 9 % of the divalent dye P2 and approximately 3 % of P3 and P4. In a fixed volume of PBS release of the dye occurs until equilibrium is achieved between the ions in the bulk and those in solution. The elution profiles of P1 from different cationic bead formulations; APTA_16_, APTA_43_ and APTA_60_, were compared together (Fig. 8b). There appears to be no difference in the elution profiles, the presence of more ‘free’ binding sites with increasing APTA in the formulation does not seem to introduce competitive binding that reduces the elution rate (a phenomenon observed with drug elution from anionically-charged AMPS-based beads [13]). The rate of P1 release is very fast from all formulations and reaches the same equilibrium concentration within 60 min.

It has been demonstrated above that the elution of different compounds may be dependent on the strength of interaction between the ionic groups. Ionic attraction between multi-charged compounds in the bulk is greater than that of a monovalent compound and the energy required to release the multi-charged dyes from the beads is greater than for the monovalent dye as it is necessary to displace multiple binding sites, instead of just one, which contributes to a slower rate of elution. As the charge density of the dyes increased there was a reduction in the equilibrium concentration achieved and the total amount of each dye released. However, there appears to be a limit to this trend as with more than three available binding sites the same release profile was observed and the achieved equilibrium concentration was unchanged.

Although the interaction of a singular sulfonate group is not strong, the presence of two groups within the structure appears to affect its release (Fig. 8c). Although the types of anionic groups are the same for the dyes studied, P2 has additional hydroxyl groups directly attached to the aromatic ring in comparison to P1. This may act to enhance the negativity of sulfonate groups and can increase the strength of interaction with the polymer. In Fig. 8a the released concentration of P3 and P4 from APTA_43_ was very low and their release over time from additional formulations was not studied. It may be assumed that a similar reduction in elution rate as observed for P2 will occur with increased charge density of the beads.

## Conclusions

In this study a series of beads were produced with different levels of the cationic monomer, APTA, in their formulation. Characterisation techniques (elemental analysis, ATR-FTIR and GC) were used to confirm the conversion of materials during synthesis and identify the presence of residual materials used in sample preparation. Suspension polymerisation was shown to successfully produce uniformly spherical copolymer beads with APTA content up to 60 wt%. The synthetic process was limited above this concentration and beads prepared using 86 wt% APTA appeared fragmented with an outer skin peeling away. EWC, permeability of large molecular weight compounds and resistance to compression increased with increasing APTA content in the formulation. It was demonstrated that the cationic microspheres could bind and release anionic compounds via an ion-exchange process through interaction with the positively charged quaternary ammonium groups of the copolymer. In elution studies as the charge density of the model drugs increased there was a reduction in the equilibrium concentration achieved and the total amount eluted in a closed release system. It was observed that the monovalent dye had the fastest rate of elution in comparison to the other dyes. Its interaction with the cationic polymer was shown to be relatively weak and when released from microspheres with increased charge density there was no reduction in the rate of elution. However when using a dye with multi-binding sites there was increased interaction with the polymer, slowing the release and also a reduced rate of elution from beads with higher charge density. The drug release studies demonstrate that these systems can be engineered for different potential anionic drugs for local therapeutic delivery in clinical applications.
